# Responsiveness of the Multiple Sclerosis International Quality of Life questionnaire to disability change: a longitudinal study

**DOI:** 10.1186/1477-7525-11-127

**Published:** 2013-07-29

**Authors:** Karine Baumstarck, Helmut Butzkueven, Oscar Fernández, Peter Flachenecker, Sergio Stecchi, Egemen Idiman, Jean Pelletier, Mohamed Boucekine, Pascal Auquier

**Affiliations:** 1 EA3279, Self-perceived Health Assessment Research Unit, School of Medicine, Aix-Marseille Université, Marseille, France; 2 Melbourne Brain Centre at the Royal Melbourne Hospital, Department of Medicine, University of Melbourne, Melbourne, VIC 3010, Australia; 3 Department of Neurology, Box Hill Hospital, Monash University, Box Hill VIC 3128, Australia; 4 Institute of Clinical Neurosciences, Hospital Regional Universitario Carlos Haya, Málaga, Spain; 5 Neurological Rehabilitation Center Quellenhof, Bad Wildbad, Germany; 6 Multiple Sclerosis Unit, IRCCS Istituto delle Scienze Neurologiche, Azienda Bologna, USL, Italy; 7 Department of Neurology, Dokuz Eylül University, Izmir, Turkey; 8 Department of Neurology, Timone University Hospital, Marseille, France

**Keywords:** Multiple sclerosis, Quality of life, Outcome research, Responsiveness, MusiQoL, SF-36, Longitudinal studies

## Abstract

**Background:**

Responsiveness, defined as the ability to detect a meaningful change, is a core psychometric property of an instrument measuring quality of life (QoL) rarely reported in multiple sclerosis (MS) studies.

**Objective:**

To assess the responsiveness of the Multiple Sclerosis International Quality of Life (MusiQoL) questionnaire to change in disability over 24 months, defined by change in the Expanded Disability Status Scale (EDSS) score.

**Methods:**

Patients with MS were enrolled into a multicenter, longitudinal observational study. QoL was assessed using both the MusiQoL and the 36-Item Short-Form (SF-36) instruments at baseline and every 6 months thereafter up to month 24; neurological assessments, including EDSS score, were performed at each evaluation.

**Results:**

The 24-month EDSS was available for 524 patients. In the 107 worsened patients, two specific dimensions of MusiQoL, the sentimental and sexual life and the relationships with health care system dimensions, and ‘physical’ scores of SF-36 showed responsiveness.

**Conclusions:**

Whereas specific dimensions of MusiQoL identified EDSS changes, the MusiQoL index did not detect disability changes in worsened MS patients in a 24-month observational study. Future responsiveness validation studies should include longer follow-up and more representative samples.

## Introduction

Multiple sclerosis (MS) is a chronic inflammatory demyelinating disease of the central nervous system that affects from 1 to 8 per 100.000 young adults [1]. While the physical disability is of great importance in (MS), it is now well-recognized that it does not reflect all the aspects that patients consider important in their life. It is only one aspect of a person’s experience, and MS is associated with a significant decreased health-related quality of life (QoL) [2,3]. So, monitoring QoL is important as patients report that many aspects of the disease affect their QoL. Although QoL instruments potentially capture many effects of MS that are not reflected in the disability assessment based on the Expanded Disability Status Scale (EDSS) [2,4-6], it is a complex task to validate their relationship to the disease in question. One potential method of validation is to assess the association between disability change and change in the QoL instrument, termed ‘responsiveness’ or ‘sensitivity to change’. This property, defined as the ability to detect a meaningful change, is a core psychometric property of a measuring instrument [7-9]. However, examination of responsiveness requires longitudinal data collection and is, therefore, rarely reported in MS QoL studies.

The MS International QoL (MusiQoL) questionnaire is a well-validated MS-specific, self-administered, multidimensional, patient-based QoL instrument [10] initially co-developed and validated in 14 languages. In the initial MusiQoL validation study, responsiveness was assessed in a small sample size at day 21 [10].

The present study sought: i) to assess the responsiveness of MusiQoL to changes in disability over 24 months in patients with MS, defined by changes in the EDSS score [11,12]; ii) to compare the responsiveness between MusiQoL and a generic instrument (SF-36).

## Methods

### Study design and setting

This was a multicenter, multiregional, longitudinal study carried out at 32 centers in 12 countries (see Additional file 1: Table S1): Argentina (3 centers), Australia (5), Austria (3), Germany (3), Spain (1), France (2), Israel (5), Italy (5), Norway (1), Turkey (1), the United Kingdom (2), and the United States (1). This was a study investigator-initiated.

### Standard protocol approvals, registrations, and patient consent

This study (ClinicalTrials.gov identifier: NCT00702065) was performed in accordance with the Declaration of Helsinki and all applicable regulatory authority requirements and national laws. Written informed consent from patients was obtained prior to any study procedures.

### Patients

Patients were eligible for the study if they were aged 18 years or older, had any form of definite MS (2001 or 2005 McDonald [13] and/or Poser criteria [14]), had an EDSS score ≤7.0, were with or without treatment including disease-modifying drugs, and were able to complete the QoL questionnaires independently. Patients were eligible regardless of approved MS treatment received. Patients were excluded if they were in clinically isolated syndrome status, were receiving or assigned to receive any investigational drug or undergo any experimental procedure during the study, or had a major medical or psychiatric illness (including dementia). All therapeutic decisions during the study were at the discretion of the treating physician.

### Assessments and data collection

Clinical data and MS treatments were recorded by the physician using an electronic case report form. QoL data were collected using paper questionnaires, which were completed by patients in the waiting room of the centers. Five assessments were performed in total: at the time of enrollment (baseline evaluation), and every 6 months thereafter up to month 24.

#### Demographic and clinical data

Demographic data and history of MS, including MS course, number of relapses within the previous 24 months, date of disease onset, and all ongoing MS treatments, were collected at baseline. At baseline and subsequent evaluations, patients underwent a neurological evaluation, including and MS course. Neurological disability status was assessed using a neurologist-rated Expanded Disability Status Scale (EDSS) including eight Functional Systems scores (FS) [15]: pyramidal, cerebellar, brainstem, sensory, bowel and bladder, visual, cerebral, and other. Each of the FSS is an ordinal clinical rating scale ranging from 0 to 5 or 6. The EDSS is an ordinal clinical rating scale ranging from 0 (normal neurologic examination) to 10 (death due to MS). Treatment and clinical relapses (defined as the appearance of a new symptom or group of symptoms, or the worsening/reappearance of old symptoms lasting at least 24 hours, in the absence of fever and preceded by stability or improvement for at least 30 days) were also recorded.

#### Quality of life

QoL was assessed at baseline and at all subsequent assessments using two questionnaires, the MusiQoL and the Short Form 36 (SF-36).

The MusiQoL questionnaire comprises 31 questions in 9 dimensions (subscales): activities of daily living (ADL, 8 items), psychological well-being (PWB, 4), symptoms (SPT, 4), relationships with friends (RFr, 3), relationships with family (RFa, 3), sentimental and sexual life (SSL, 2), coping (COP, 2), rejection (REJ, 2), and relationships with healthcare system (RHCS, 3). The index score is computed as the mean of these subscale scores. All 9 dimensions and the index score are linearly transformed and standardized on a 0 to 100 scale, where 0 indicates the worst possible level of QoL and 100 indicates the best level. Differential item functioning analysis were performed in the initial validation study showing satisfactory results across countries [10]. The SF-36 (version 2) questionnaire comprises 36 items grouped into 8 subscales; two standardized summary scores are also derived: physical component summary (PCS) and mental component summary (MCS). The PCS and MCS scores are norm-based, using a linear T-score transformation with a mean of 50 and a standard deviation of 10. Scores range from 0 to 100, with higher values indicating better QoL.

### Worsened and non-worsened patients

Patients were defined as worsened or non-worsened based on change in EDSS score from baseline to month 24. The worsened patient group was characterised by a 24-month increase in the EDSS score by one point if the baseline EDSS score was less than 5.5, or an increase in the EDSS score by half a point if the baseline EDSS score was between 5.5 and 7.0. All other patients were defined as non-worsened [16,17].

### Study outcomes

The population analysed included all patients with a MusiQoL assessment at baseline and at a subsequent assessment, and EDSS score at baseline and at the same subsequent assessment. The primary outcome was the change in MusiQoL scores between baseline and month 24 [18]. Secondary outcomes were: change in MusiQoL scores between baseline and each of months 6, 12, and 18; changes in MusiQoL subscale scores and SF-36 subscale and summary scores between baseline and each subsequent visit.

### Statistical analysis

Comparisons between the two groups, worsened or non-worsened patients, were performed using a chi-square test for categorical variables or a Mann–Whitney test for quantitative variables. The effect size (ES) was calculated as the mean change in QoL (MusiQoL and SF-36) scores between baseline (BL) and i-month (Mi: M6, M12, M18, and M24) divided by the standard deviation of the baseline score [8]. An ES of at least 0.2 is recommended as the standard for supporting a minimal sensitivity to change. ES of 0.2, 0.5 and 0.8 is considered as small, moderate and large change respectively [18,19]. To quantify how the responsiveness differs between the worsened and non-worsened patients, standardized variations between i-month and BL were calculated as the final QoL value minus the initial QoL value divided by the initial QoL value for each patient and for each QoL score. Standardized variations were compared between worsened or non-worsened patients using Mann–Whitney tests. A two-sided paired t-test at 5% significance level was used to assess whether the change in the QoL scores from baseline to month 24 was significantly different from zero. To assess change over time of QoL dimensions in each groups (worsened or non-worsened groups), mixed linear modeling was performed using unstructured covariance matrix after adjusted for covariates: gender, age, marital status, employment status, education level, and disease duration. The annualized relapse rate was estimated using a Poisson regression model.

## Results

### Patient characteristics

**Figure 1:**
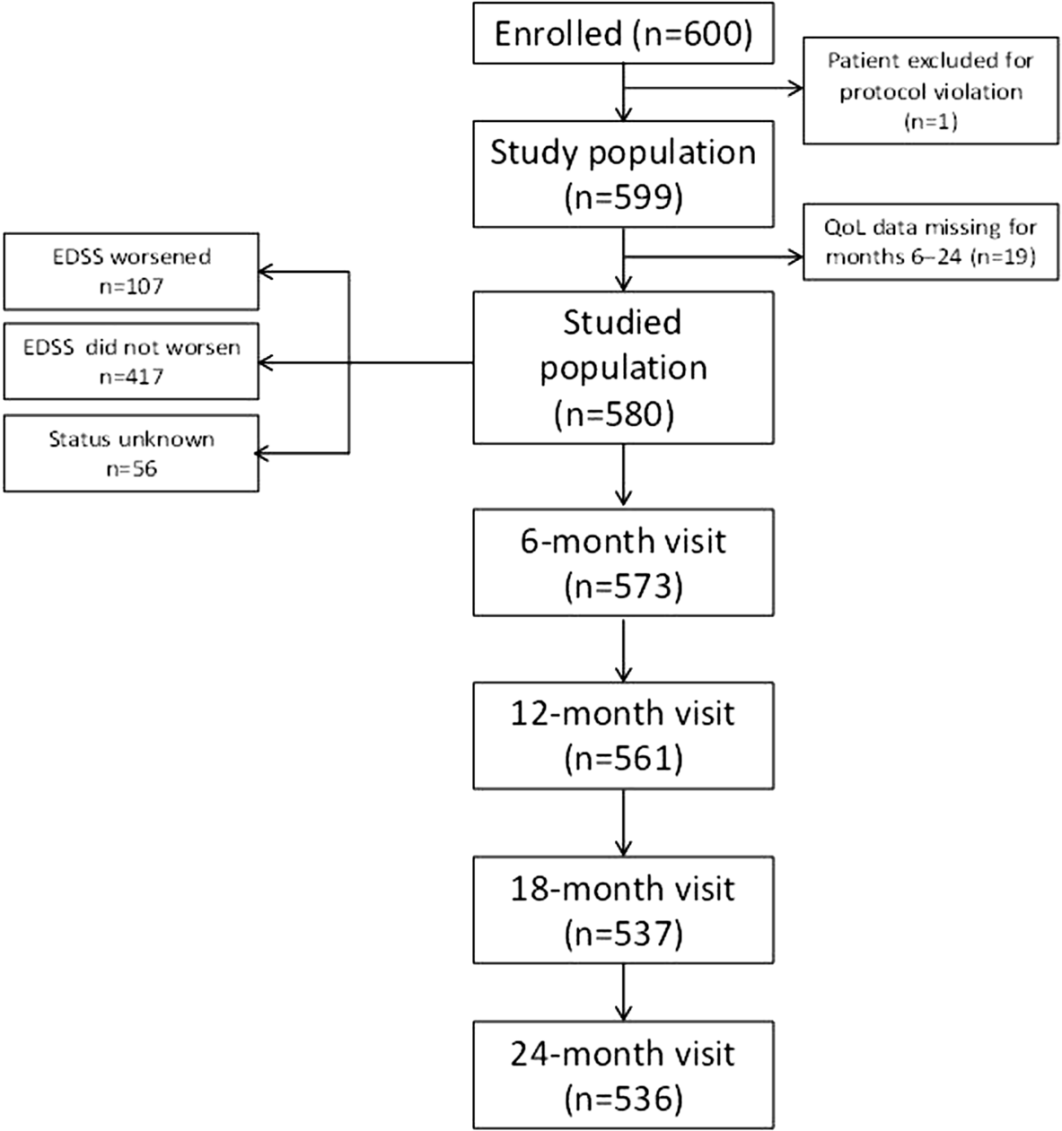
Patient disposition.

In total, 600 patients were enrolled from 12 countries between November 2007 and October 2010; 1 patient was excluded because of a protocol violation (eligibility requirements not met) and 19 patients were excluded due to missing QoL data at months 6, 12, 18, and 24 (Figure 1). The final sample comprised 580 patients. The 24-month EDSS was available for 524 of 536 patients assessed at 24-month. A total of 417 (79.6%) patients were defined as non-worsened and 107 (20.4%) patients were defined as worsened. The baseline demographic characteristics differed between worsened and non-worsened subjects, with worsened patients on average being older, with higher baseline EDSS scores and a higher proportion of primary and secondary progressive MS than non-worsened patients (Table 1). At inclusion, 551 (95.0%) patients were taking disease-modifying drugs. Over the course of the study, 366 patients (63.1%) were relapse free. In total, 204 patients had ≥1 relapse (data were unavailable for 10 patients); of these patients, most had either one (n = 137) or two (n = 42) relapses; 4 patients had ≥5 relapses. The mean (SD) number of relapses occurring during the study was 0.54 (0.90) and the annualized relapse rate was 0.28 (95% confidence interval: 0.25, 0.31). The mean (SD) change from baseline in EDSS score was −0.03 (0.71) at month 6, -0.03 (0.86) at month 12, -0.01 (0.91) at month 18, and 0.03 (0.95) at month 24.

**Table 1:** Baseline patient demographics and disease characteristics

		**Total sample°**	Worsened patients*	Non-worsened patients*	
		**N = 580**	**N = 107**	**N = 417**	**p**
Female, n (%)		419 (72.2)	75 (70.1)	300 (71.9)	0.71
Age (years)	M (SD)	41.3 (10.2)	43.2 (10.2)	40.9 (10.1)	**0.02**
	Min, max	18, 71	19, 64	18, 69	
Marital status, n (%)	Cohabiting/married Divorced/separated/single/widowed	393 (67.8)	73 (68.2)	282 (67.6)	0.91
		187 (32.2)	34 (31.8)	73 (68.2)	
Employment status, n (%)	Employed	335 (57.8)	59 (55.1)	241 (57.8)	0.62
	Unemployed/homemaker/retired/student	245 (42.2)	48 (44.9)	176 (42.2)	
Educational level, n (%)	Elementary school	113 (19.5)	31 (28.4)	76 (18.2)	**0.03**
	College	81 (14.0)	9 (8.3)	61 (14.6)	
	High school/university	386 (66.6)	67 (63.3)	280 (67.1)	
EDSS score	M (SD)	2.9 (1.9)	3.5 (2.2)	2.8 (1.8)	**<10**^**-3**^
	Median	2.5	4.0	2.0	
	Min, max	0.0, 7.5	0.0, 7.0	0.0, 7.0	
MS course, n (%)	Relapsing-remitting	510 (87.9)	79 (73.8)	381 (91.4)	**<10**^**-3**^
	Secondary progressive	53 (9.1)	18 (16.8)	30 (7.2)	
	Primary progressive	12 (2.1)	8 (7.5)	2 (0.5)	
	Primary relapsing	5 (0.9)	2 (1.9)	4 (1.0)	
Time since first MS symptom (years)	M (SD)	10.0 (7.5)	10.2 (8.1)	9.9 (7.2)	0.98
	Min, max	0, 45	0, 45	0.0, 40	
Number of relapses in previous 2 years	M (SD)	1.3 (1.4)	1.4 (1.6)	1.3 (1.3)	0.33
	0	187 (32.2)	37 (34.6)	135 (32.4)	
	1	185 (31.9)	27 (25.2)	137 (32.9)	
	2	123 (21.2)	23 (21.5)	87 (20.9)	
	> = 3	85 (14.7)	20 (18.7)	58 (13.9)	

M (SD), mean (standard deviation).

EDSS, Expanded Disability Status Scale; MS, multiple sclerosis.

° the 24-month EDSS was not available for 56 individuals.

* defined as worsened or non-worsened based on change in EDSS score from baseline to month 24 according to the definition of Lublin [Lublin 1996] and Kappos [Kappos 2004].

Bold values: p-value < 0.05.

### Responsiveness of MusiQoL and SF-36 at month 24 in worsened patients

In worsened patients, two MusiQoL dimensions, sentimental and sexual life and relationships with healthcare system, had an ES higher than 0.20 at month 24 (Table 2), indicating an association with disability change. The standardized variation of the activity of daily living dimension and the sentimental and sexual life dimension of MusiQoL were statistically different between worsened group and non-worsened groups. The ES of the physical component summary and two dimensions of the SF-36 (physical functioning and bodily pain) were higher than 0.20. For the SF-36 physical component summary score, and 3 dimensions of SF-36 (physical functioning, role physical, and bodily pain), the standardized variations were statistically different between worsened and non-worsened patients (Tables 2 and 3).

**Table 2:** Responsiveness of MusiQoL and SF-36 in the 107 worsened patients

	**Baseline**	**Month 6**			**Month 12**			**Month 18**			**Month 24**		
**MusiQoL**	**M (SD)**	**M (SD)**	**D**^**M6-BL**^	**ES**	**M (SD)**	**D**^**M12-BL**^	**ES**	**M (SD)**	**D**^**M18-BL**^	**ES**	**M (SD)**	**D**^**M24-BL**^	**ES**
ADL	50.0 (24.4)	46.9 (25.2)	−3.33	−0.14	49.4 (26.1)	−1.04	−0.04	47.1 (26.9)	−3.04	−0.12	46.8 (28.2)	−3.53	−0.14
PWB	62.6 (22.1)	62.5 (23.0)	−0.56	−0.02	64.8 (22.5)	2.04	0.09	61.7 (23.2)	−1.33	−0.06	64.3 (23.9)	1.67	0.08
SPT	64.7 (21.3)	68.1 (21.6)	3.54^*^	0.16	69.2 (20.4)	4.29^**^	0.20	65.3 (21.6)	0.22	0.07	67.2 (21.3)	2.16	0.10
RFr	62.2 (25.0)	59.3 (24.6)	−2.72	−0.11	60.3 (24.0)	−1.33	−0.05	64.4 (23.4)	1.55	0.06	60.3 (23.6)	−1.67	−0.07
RFa	74.6 (22.5)	73.2 (21.9)	−1.35	−0.06	69.7 (25.3)	−4.46	**−0.20**	73.4 (23.0)	−1.83	−0.08	72.6 (23.2)	−2.17	−0.10
SSL	63.3 (29.4)	59.5 (28.7)	−3.65	−0.12	57.7 (29.3)	−4.89*	−0.16	54.3 (30.5)	−9.62^***^	**−0.32**	56.1 (30.6)	−6.38*	**−0.21**
COP	66.1 (27.0)	66.7 (26.1)	0.36	0.01	67.2 (26.1)	0.12	0.00	67.3 (27.2)	1.14	0.04	64.5 (28.6)	−0.73	−0.03
REJ	78.7 (27.7)	78.1 (24.4)	−0.36	−0.01	79.4 (23.8)	0.61	0.02	78.1 (24.8)	−0.38	−0.01	78.2 (24.8)	−0.12	−0.00
RHCS	86.8 (14.8)	82.6 (19.1)	−4.49^**^	**−0.30**	82.9 (17.2)	−3.96^**^	**−0.27**	81.9 (20.4)	−4.93^**^	**−0.33**	80.7 (19.7)	−5.98^***^	**−0.40**
Index	67.0 (13.1)	65.8 (13.9)	−1.12	−0.09	66.1 (13.1)	−1.08	−0.08	65.7 (13.8)	−2.46^**^	−0.19	65.2 (15.0)	−2.26	−0.17
**SF-36v2**	**M (SD)**	**M (SD)**	**D**^**M6-BL**^	**ES**	**M (SD)**	**D**^**M12-BL**^	**ES**	**M (SD)**	**D**^**M18-BL**^	**ES**	**M (SD)**	**D**^**M24-BL**^	**ES**
PF	48.4 (28.5)	45.2 (29.0)	−2.95	−0.10	46.6 (29.8)	−2.19	−0.08	42.9 (30.4)	−5.37^**^	−0.19	41.1 (32.0)	−7.84^***^	**−0.28**
RP	50.4 (27.9)	50.3 (28.8)	−0.04	−0.00	50.9 (28.1)	−0.55	−0.02	45.1 (27.8)	−4.60	−0.17	46.0 (29.8)	−4.83^*^	−0.17
BP	67.6 (26.3)	64.1 (26.7)	−3.88	−0.15	64.1 (25.7)	−3.10	−0.12	61.6 (28.2)	−5.88^**^	**−0.22**	60.9 (27.3)	−7.44^***^	**−0.28**
GH	49.5 (21.2)	50.2 (18.8)	0.89	0.04	47.4 (20.1)	−2.83	−0.13	49.3 (20.0)	−0.59	−0.03	47.9 (21.4)	−2.27	−0.11
Vi	43.5 (18.3)	45.7 (20.2)	2.08	0.11	44.6 (19.6)	1.04	0.06	43.1 (19.4)	−0.58	−0.03	44.1 (20.3)	0.58	0.03
SF	65.8 (24.1)	64.9 (25.5)	−1.09	−0.04	65.3 (27.4)	−0.25	−0.01	65.0 (25.5)	−0.98	−0.04	61.9 (28.7)	−3.85	−0.16
RE	53.0 (14.9)	54.8 (12.5)	1.29	−0.09	52.5 (13.3)	−0.59	−0.04	52.8 (13.7)	−0.80	−0.05	51.7 (14.4)	−1.94	−0.13
MH	65.4 (18.7)	66.8 (18.0)	0.98	0.05	64.6 (20.5)	−1.04	−0.06	64.3 (18.2)	−1.85	−0.10	63.2 (20.4)	−3.12	−0.17
PCS	39.6 (9.9)	38.1 (9.8)	−1.19	−0.12	38.9 (9.5)	−0.93	−0.09	37.4 (10.6)	−2.13^**^	**−0.22**	37.3 (11.1)	−2.59^***^	**−0.26**
MCS	44.9 (8.6)	46.2 (8.5)	1.30	0.15	45.1 (8.6)	0.50	0.06	45.1 (8.1)	0.16	0.02	45.1 (8.9)	−0.11	−0.01

MusiQoL Multiple Sclerosis Quality of Life questionnaire.

ADL activity of daily living, PWB psychological well-being, SPT symptoms, RFr relationships with friends, RFa relationships with family, SSL sentimental and sexual life, COP coping, REJ rejection, RHCS relationships with health care system.

SF-36v2 Short Form 36 version 2.

PF physical functioning, RP role physical, BP bodily pain, GH general health, Vi vitality, SF social functioning, RE role-emotional, MH mental health, PCS physical component summary, MCS mental component summary.

M (SD), mean (standard deviation).

D^Mi-BL^: difference mean between the score of the evaluation time and the baseline score (a positive difference indicates a QoL improvement and a negative difference indicates a QoL deterioration), ^*^p < =0.05, ^**^p < =0.01, ^***^p < =0.001.

ES, effect size; bold values indicate effect sizes > = 0.20.

**Table 3:** Responsiveness of MusiQoL and SF-36 in the 417 non-worsened patients

	**Baseline**	**Month 6**				**Month 12**				**Month 18**				**Month 24**			
**MusiQoL**	**M (SD)**	**M (SD)**	**D**^**M6-BL**^	**ES**	**p**^**W/NW**^	**M (SD)**	**D**^**M12-BL**^	**ES**	**p**^**W/NW**^	**M (SD)**	**D**^**M18-BL**^	**ES**	**p**^**W/NW**^	**M (SD)**	**D**^**M24-BL**^	**ES**	**p**^**W/NW**^
ADL	62.8 (24.7)	64.7 (25.7)	1.97^**^	0.08	0.065	65.2 (24.6)	2.61^***^	0.11	0.109	65.0 (26.1)	2.09^***^	0.08	*0*.*035*	63.4 (26.4)	0.91	0.04	*0*.*017*
PWB	62.3 (23.8)	63.7 (24.7)	1.63	0.07	0.187	65.8 (23.7)	3.63^***^	0.15	0.410	65.6 (25.2)	3.21^***^	0.13	*0*.*029*	66.9 (25.0)	4.76^***^	**0**.**20**	0.145
SPT	69.4 (22.3)	70.2 (22.3)	0.83	0.04	0.196	71.2 (21.0)	1.58	0.07	0.140	71.1 (23.0)	1.70	0.08	0.694	69.8 (23.2)	1.45	0.01	0.441
RFr	59.1 (29.0)	58.1 (27.8)	−0.19	−0.01	0.601	58.6 (28.5)	−0.19	−0.01	0.937	58.1 (28.1)	−0.74	−0.03	0.301	58.8 (27.9)	−0.20	−0.01	0.754
RFa	72.5 (27.7)	71.5 (28.2)	−0.33	−0.01	0.136	69.9 (28.6)	−2.26	−0.08	0.362	69.6 (28.8)	−2.97^*^	−0.11	0.792	70.0 (29.7)	−2.29	−0.08	0.803
SSL	63.6 (29.7)	62.9 (29.9)	−0.39	−0.01	0.383	62.4 (30.7)	−1.12	−0.04	0.237	62.4 (30.3)	−1.91	−0.06	*0*.*010*	63.2 (30.6)	−0.03	−0.00	*0*.*043*
COP	62.1 (29.6)	64.8 (28.7)	3.12^**^	0.10	0.149	65.3 (29.1)	3.49^**^	0.12	0.370	65.6 (28.7)	3.65^**^	0.12	0.879	65.5 (28.2)	3.20^*^	0.11	0.467
REJ	82.6 (23.9)	84.2 (23.5)	1.88^*^	0.08	0.242	84.2 (22.8)	1.68	0.07	0.172	81.9 (24.4)	−0.76	−0.03	0.749	82.3 (23.4)	−0.22	−0.01	0.715
RHCS	85.2 (19.4)	84.7 (19.9)	−0.60	−0.03	0.138	83.5 (20.6)	−1.71	−0.09	0.517	83.5 (19.6)	−2.13^*^	−0.11	0.603	83.0 (18.8)	−2.18^*^	−0.11	0.219
Index	68.5 (14.8)	69.3 (15.0)	0.90	0.06	0.160	69.4 (15.4)	0.75	0.05	0.365	69.1 (16.4)	0.27	0.02	*0*.*014*	69.4 (15.6)	0.34	0.02	0.068
**SF-36v2**	**M (SD)**	**M (SD)**	**D**^**M6-BL**^	**ES**	**p**^**W/NW**^	**M (SD)**	**D**^**M12-BL**^	**ES**	**p**^**W/NW**^	**M (SD)**	**D**^**M18-BL**^	**ES**	**p**^**W/NW**^	**M (SD)**	**D**^**M24-BL**^	**ES**	**p**^**W/NW**^
PF	65.2 (27.7)	65.5 (27.4)	0.11	0.00	0.472	65.9 (28.0)	0.83	0.03	0.086	66.8 (29.0)	1.11	0.04	<*10*^-*3*^	65.4 (29.5)	0.30	0.01	<*10*^-*3*^
RP	60.2 (29.6)	62.0 (28.5)	1.63	0.06	0.733	63.1 (28.7)	3.08^*^	0.10	0.233	63.2 (28.8)	2.77^*^	0.09	*0*.*016*	63.1 (28.6)	3.06^*^	0.10	*0*.*001*
BP	66.8 (27.2)	66.9 (27.6)	0.08	0.00	0.628	67.5 (25.5)	0.36	0.01	0.866	68.2 (26.7)	1.12	0.04	0.139	67.0 (26.5)	0.28	0.01	*0*.*044*
GH	54.7 (22.4)	54.6 (22.4)	−0.02	−0.01	0.766	54.9 (22.0)	0.10	0.00	0.107	53.1 (23.0)	−1.35	−0.06	0.646	52.7 (23.7)	−2.03^*^	−0.09	0.859
Vi	48.8 (22.3)	49.3 (23.0)	0.72	0.03	0.667	50.0 (22.4)	1.22	0.05	0.779	50.4 (22.8)	1.54	0.07	0.681	48.7 (23.2)	0.09	0.00	0.965
SF	69.0 (26.6)	71.0 (27.0)	2.12	0.08	0.330	70.4 (27.2)	1.29	0.05	0.531	70.6 (26.2)	1.71	0.06	0.356	70.3 (26.3)	1.33	0.05	0.117
RE	56.1 (13.7)	56.0 (12.7)	−0.08	−0.00	0.392	56.5 (12.0)	0.27	0.02	0.251	56.2 (11.5)	−0.06	−0.00	0.466	56.2 (12.3)	0.04	0.00	0.124
MH	63.9 (20.5)	64.4 (21.3)	0.65	0.03	0.853	65.3 (19.5)	1.25	0.06	0.383	64.2 (20.6)	0.29	0.01	0.132	64.4 (20.7)	0.41	0.02	0.069
PCS	44.1 (10.0)	44.2 (10.1)	0.11	0.01	0.702	44.4 (10.1)	0.41	0.04	0.349	44.6 (10.5)	0.54	0.05	*0*.*003*	44.1 (10.5)	0.05	0.01	<*10*^-*3*^
MCS	43.8 (8.9)	44.2 (9.0)	0.38	0.04	0.260	44.3 (8.8)	0.48	0.05	0.773	44.0 (9.0)	0.04	0.00	0.973	44.0 (9.2)	0.20	0.02	0.700

MusiQoL Multiple Sclerosis Quality of Life questionnaire.

ADL activity of daily living, PWB psychological well-being, SPT symptoms, RFr relationships with friends, RFa relationships with family, SSL sentimental and sexual life, COP coping, REJ rejection, RHCS relationships with health care system.

SF-36v2 Short Form 36 version 2.

PF physical functioning, RP role physical, BP bodily pain, GH general health, Vi vitality, SF social functioning, RE role-emotional, MH mental health, PCS physical component summary, MCS mental component summary;

M (SD), mean (standard deviation).

D^Mi-BL^: difference mean between the score of the evaluation time and the baseline score (a positive difference indicates a QoL improvement and a negative difference indicates a QoL deterioration), ^*^p < =0.05, ^**^p < =0.01, ^***^p < =0.001.

ES, effect size; bold values indicate effect sizes > = 0.20.

p^W/NW^, p-value of the comparison of standardized variation (final value minus the initial value divided by the initial value) between worsened and non-worsened individuals; italic values indicate p-value <0.05.

### Responsiveness of MusiQoL and SF-36 at months 6, 12, and 18 in worsened patients

A small ES was obtained for the relationships with healthcare system dimension at all assessments, for relationships with family at month 12, and for sentimental and sexual life at month 18 (Table 2). The physical component summary and the bodily pain dimension of SF-36 at month 18 showed some responsiveness in worsened patients, lower than −0.20 (Table 2). At month-18, the standardized variations were statistically different between worsened and non-worsened patients for the index and 3 dimensions of MusiQoL (activity of daily living, psychological well-being, and sentimental and sexual life), and for the physical component summary and 2 dimensions of SF-36 (physical functioning and role physical) (Tables 2 and 3). In the earliest evaluation visits (6 and 12 months), neither subscale scores nor composite scores of SF-36 were associated with EDSS changes.

### Changes in QoL scores over the time

In the worsened patients, dimensions showed significant change over time after adjustment for covariates: activity of daily living, relationships with healthcare system, and sentimental and sexual life (respective p-values: 0.04, <10^-3^, and 0.001) for MusiQoL, and physical functioning, role physical, bodily pain, and the physical component summary (all p-values: < 0.001) for SF-36. In the non-worsened group, 4 dimensions of MusiQoL showed significant changes (psychological well-being relationships with family, relationships with healthcare system, and coping; respective p-values:<10^-3^, 0.04, 0.02, and 0.03), and 2 of SF-36 (role physical and general health, p = 0.03 and 0.02 respectively). These significant changes were in accordance with the calculated ES, showing the higher ES for these dimensions.

## Discussion

This is the first study that reports details of the responsiveness of a MS-specific QoL questionnaire, the MusiQoL, in a large longitudinal study. Compared to the international and European MS populations [10,20], our patients presented a lower sex-ratio (0.38 versus 0.41 and 0.60, respectively), a less severe disability profile (EDSS median 2.4 versus 3.2 and 4.1, respectively), and a lower proportion of secondary progressive MS (9% versus 21% and 36%, respectively). These disparities may partially explain the higher QoL scores reported by this population compared to others.

QoL scores of both the MS-specific and generic instruments were weakly responsive to EDSS change between baseline and month 24. However, while the SF-36 detected changes only almost from the ‘physical-like’ dimensions (physical functioning, bodily pain, and psychical component summary), the MusiQoL identified EDSS changes from ‘non-physical’ dimensions, such as relationships with healthcare system and sentimental and sexual life. This weak responsiveness should not be explained by the short nature of some scales (because high ES were found for short dimensions), neither by the conceptual basis of the questionnaire elaboration, that was clearly defined in the initial publication [10]. Standardized variations of the MusiQoL differed significantly between worsened and non-worsened patients for ‘psychological-like’ dimensions, such as psychological well-being and sentimental and sexual life. Whereas the SF-36 seems to capture mostly QoL deficits due to physical changes, MusiQoL, via specific dimensions such as sentimental and sexual life, detects emotional and social aspects of the MS disease process often underestimated by clinical judgment. Lastly, the MusiQoL index (at month-18) detected a global change whereas only the physical composite of the SF-36 and not the mental composite score demonstrated responsiveness to EDSS change.

In our analysis, we did not find convincing differences of responsiveness between MusiQoL and SF-36 subscales. These findings were not in line with previous reports indicating that specific instruments are potentially more responsive and more sensitive for detecting and quantifying small disease state changes than generic health status measures [21,22], observations that have also been specifically reported in MS [23,24]. Anyway, we consider the consensus that both general and specific tools are needed in order to provide a comprehensive assessment of global health and disease specific issues is still valid [22]. Generic instruments are generally used to compare QoL across different populations, while disease-specific instruments focus on particular health problems and are more sensitive for detecting and quantifying small changes. In MS clinical practice, MS-specific questionnaires are more appropriate due to a better ability to discern QoL differences in patients than the SF-36. Authors demonstrated that modifying existing measures by adding items may not be useful in improving the measurement properties of an instrument [25].

The responsiveness of QoL instruments to EDSS change reported in this study can be considered low whatever the evaluation times and the nature of the questionnaire, generic or MS-specific. Capacity to detect association with EDSS change was limited by the duration of our study, in that 24-month follow-up was perhaps too short to detect associations, in particular as a lower number of patients than expected “worsened” in comparison to historical data [26], which reduced the power of the study to detect responsiveness.

Additionally, the relapse rate was low throughout the study, indicating a population with relatively low disease activity. Future responsiveness validation studies should include longer follow-up or, perhaps, focus on patients who would be expected to have more rapidly worsening disability, for example patients with progressive forms of MS. We chose not to do this during the planning of the study because we wished to investigate QoL responsiveness across a wide range of patients with differing disease severities. Of course, one might not expect large effect sizes in responsiveness studies of this kind, because QoL instruments and physical disability rating scales do not contain highly overlapping information, and the premise of our validation study was the detection of any association between QoL change and EDSS change, as evidence for relevance of the QoL instruments in MS, rather than expectation of strong associations. In general, the relationships between QoL instrument change and physical disability progression is likely to be weak because, over time, disability change is associated with new coping strategies and adjustment, leading to response shifts [27,28]. This is especially relevant for global QoL scores and affective-emotional dimensions. Response shift is now known to affect adaptation to a wide degree of health conditions, including MS [29], the result of an adaptive response to a changed health status, and as such is viewed as a positive phenomenon.

Given the availability of many QoL instruments, little research has surprisingly been conducted to test the responsiveness of QoL tools in MS. Comparisons with responsiveness indices for other QoL instruments in the literature are difficult without a direct head-to-head comparison because the situations in which they were tested are not comparable. Some features should be discussed: i) the study design: two approaches have been recommended for assessing responsiveness [30], from observational studies where patients are treated with usual medical care [23,24,31,32], and more often from clinical studies of interventions where the intervention is expected to produce a change in health [23,31-33]; ii) the time between the 2 evaluations differ: 6 to 12 months [32], 2 years [34], 5 years [24], or including a larger window (6–18 months) [31] in the longitudinal studies, and from few weeks to months in the clinical studies, depending on the expected time to show the intervention effect [23,31,33,35,36]; iii) the methods to determine the subject’s health status change: from ‘transition questions’ where the patient [36,37], or the physician, or both [31] are asked to compare the current status to an earlier time point, rarely from a EDSS deterioration, and sometimes absence of precise definition [23]; iv) the sample size is often small [31] or not provided [23]. These disparities complicate the choice of scales for studies, which often involves extrapolating findings from studies in different samples.

The representativeness of our sample should be discussed. Compared with the most important longitudinal studies that parallel the present study [38-40], our patients were younger or older (mean ages of 42 [38], 44 [39], and 34 years [40], respectively), had less severe baseline disability statuses (mean EDSS score of 4.1, 5.1, and 2.9, respectively), had a sex-ratio of 3:1 (4:1, 2:1, and 2.5:1, respectively), and presented a low number of worsened patients while Benito-Leon [41] identified 30% having clinical progression of disability over an identical follow-up period. The patients were consecutive outpatients assessed in a context of MS treatment monitoring, often presented a stable disease. These specificities may partially be explained by a selection bias due to restrictive inclusion criteria, which excluded the most severe individuals (baseline EDSS scores higher than 7.0) and included a high proportion of patients treated with disease modifying therapies (95%).

## Conclusion

This study showed that the MusiQoL may be incorporated in longitudinal studies to detect quality of life changes in MS patients. The MusiQoL index score and specific MusiQoL dimensions, such as relationships with health care system or sentimental and sexual life, were moderately responsive to disability change in MS patients over the course of this 24-month study. The present study could also inform the longitudinal design of future QoL responsiveness studies, in particular in relation to the relatively small proportion of patients exhibiting EDSS worsening over 24 months of follow-up. Future responsiveness validation studies should include longer follow-up.

## Competing interests

KB, EI, MB, and PA report no competing interests. HB scientific advisory boards for Biogen Idec, Novartis, Merck Serono, and Sanofi; conference travel support from Novartis, Biogen Idec, Merck Serono, and Sanofi; serves on steering committees for trials conducted by Merck Serono, Biogen Idec, and Novartis; research support from Merck Serono, Novartis, and Biogen Idec in his capacity as honorary chair of the MSBase Foundation; editorial board of *Multiple Sclerosis International* and *Multiple Sclerosis and Related Disorders*; current recipient of a National Health and Medical Research Council (NHMRC) Career Development Award (628856), NHMRC Project Grants (566513, 628799, 1009757), NHMRC Centre of Excellence Award (1001216), an Australian Research Council Linkage Grant (LP110100473)RG, and a National MS Society (USA) Project Grant (RG3850A3/1). OF honoraria for serving as a consultant in advisory boards, or chair or speaker in meetings; and for participation in clinical trials and other research projects promoted by Biogen Idec, Bayer-Schering, Merck Serono, Sanofi, Teva, and Novartis. PF speaker fees and honoraria from Bayer Schering, Biogen idec, Sanofi, Merck Serono, Novartis, and Almirall; research grants from Bayer Schering, Sanofi, and Merck Serono. SS speaker fees and honoraria from Merck Serono. JP scientific advisory boards and steering committees for trials conducted by Merck Serono, Bayer Schering, Biogen Idec, Sanofi, Teva Neuroscience, and Novartis. Received compensation and/or his research work has been funded, entirely or in part, by a grant to his university. The grant agreement requires that the name of the funding entity and the purpose of the grant may not be disclosed. The funding entity is a governmental organization.

## Authors’ contributions

Conception and design: HB, OF, PF, SS, EI, JP, PA. Study coordination: PA. Inclusion and clinical data collection: HB, OF, PF, SS, EI, JP, PA. Analysis of data: KB, MB, PA. Interpretation of data: KB, MB, PA. Drafting and writing of manuscript: KB, PA. Revision of manuscript: HB, OF, PF, SS, EI, JP, MB. All authors read and approved the final manuscript.

## Supplementary Material

Additional file 1: Table S1 Investigators and centers. Click here for file
